# Gene Expression of Aquaporins (AQPs) in Cumulus Oocytes Complex and Embryo of Cattle

**DOI:** 10.3390/ani13010098

**Published:** 2022-12-27

**Authors:** Julieth M. Petano-Duque, Rafael E. Castro-Vargas, Juan S. Cruz-Mendez, Kelly J. Lozano-Villegas, María P. Herrera-Sánchez, Heinner F. Uribe-García, Juan S. Naranjo-Gómez, Rafael J. Otero-Arroyo, Iang S. Rondón-Barragán

**Affiliations:** 1 Research Group in Immunobiology and Pathogenesis, Laboratory of Immunology and Molecular Biology, Faculty of Veterinary Medicine and Zootechnics, Universidad del Tolima, Santa Helena Highs, Ibagué 730006299, Tolima, Colombia; 2 Poultry Research Group, Laboratory of Immunology and Molecular Biology, Faculty of Veterinary Medicine and Zootechnics, Universidad del Tolima, Santa Helena Highs, Ibagué 730006299, Tolima, Colombia; 3 Grupo de Investigación en Reproducción y Mejoramiento Genético Animal, Facultad de Ciencias Agropecuarias, Universidad de Sucre, Sincelejo 700001, Sucre, Colombia; 4 Laboratorio de Reproducción Animal, Corporación de Ciencias Biotecnológicas, Embriotecno, Montería 230029, Córdoba, Colombia

**Keywords:** aquaporins, embryos, cumulus oocyte complex, *B. indicus*, *B. taurus*

## Abstract

**Simple Summary:**

Aquaporins are membrane channels that allow for the movement of water and solutes in cells. They have been reported to play crucial roles in mammalian early development and cryopreservation processes. However, there are few studies focused on the characterization of aquaporins in cumulus oocyte and embryo complexes of cattle. Moreover, no studies have been carried out on Brahman, Holstein, Gir and Romosinuano, important bovine breeds in milk and meat production. Therefore, the objective of this study was to evaluate their presence, transcript level and possible functions in the cumulus oocyte complex of the Brahman, Holstein, Gir and Romosinuano breeds and in embryos from five bovine crosses. Aquaporins 1–12 were found in both cumulus oocyte complexes and embryos, and we found possible parental effects on the expression of aquaporins 6 and 12b in cumulus oocyte complexes and aquaporins 4, 8 and 9 in embryos. This allows one to evidence possible functions in the early development of the Brahman, Holstein, Gir and Romosinuano bovine breeds.

**Abstract:**

Aquaporins (*AQPs*) are proteins with various functions related to proper cell function and early development in mammals. The aim of this study was to evaluate the presence of *AQPs* and determine their mRNA levels in the cumulus oocyte complex (COC) of four bovine breeds and in blastocysts of five bovine crosses. Grade I, II and III COCs were collected by ovum pick up from non-lactating heifers of the Brahaman, Holstein, Gir and Romosinuano breeds. Embryos were produced in vitro up to the blastocyst stage of the bovine ♀Gir × ♂Holstein, ♀Holstein × ♂Gir, ♀Brahman × ♂Holstein, ♀Holstein × ♂Brahman, and ♀Romosinuano × ♂Holstein crosses. mRNA expression of *AQP1*-*AQP12b* was estimated in COC and embryos by real-time-PCR. The presence of the twelve *AQPs* in the COCs and bovine embryos was established. Additionally, significant differences were determined in the expression of *AQP6* and *AQP12b* in COCs, as well as in transcripts levels of *AQP4*, *AQP8* and *AQP9* from bovine embryos. Gene expression of *AQPs* in COCs and bovine embryos is consistent with the previously described biological functions. This is the first report of *AQPs* in COC of Gir, Brahman, Holstein and Romosinuano and embryos of five crossbreeds between *Bos indicus* and *B. taurus*.

## 1. Introduction

Aquaporins (*AQPs*) are a family of conserved integral transmembrane proteins considered fundamental for the correct function of cells such as water transport (i.e., fluid secretion, fluid absorption and cell volume regulation), cell adhesion, cell migration, cell proliferation and cell differentiation [1,2,3]. Additionally, *AQPs* may be involved in inflammatory processes, as regulators of the host’s innate defense at the cell membrane level [4].

In mammals, *AQPs* are expressed in leukocytes, adipocytes, kidneys, reproductive system tissues, lungs, exocrine glands, eyes and gastrointestinal organs, in which some *AQPs* are involved in fluid transport [1]. They are classified into three subfamilies according to sequence similarity and substrate preference [3]. The first group is orthodox *AQPs*, which are water-selective and include *AQP0, AQP1, AQP2, AQP4, AQP5, AQP6* and *AQP8*; the second group is aquaglyceroporins, which are permeable to glycerin, urea and water and include *AQP3, AQP7, AQP9* and *AQP10*; and the third group of super aquaporins is permeable to water and includes *AQP11* and *AQP12* [3,5]. Furthermore, another category of *AQPs*, the peroxiporins, have been reported, and they can transport H_2_O_2_ across mammalian cell membranes, and this can vary between isoforms [6]. Some of the *AQPs* that can transport the H_2_O_2_ are *AQP1*, *AQP3*, *AQP5*, *AQP8*, *AQP9*, and *AQP11* [6]. 

*AQPs* can be found in the male and female reproductive systems [7]. In the female reproductive system, *AQPs* participate in cervical dilatation during gestation, early embryonic development, follicular development and the process of cavitation, where they function as conductors for the trans-trophectodermal movement of blastocele fluid (mainly water) and the polarized distribution of Na/KATPase [8,9,10]. In cattle embryos and oocytes, the function of *AQPs* has been studied during the cryopreservation process where cells dehydrate and rehydrate [11,12].

Differences have been shown regarding gene expression patterns related to reproductive features such as oocyte quality, oocyte recovery, blastocyst production and pregnancy rates, depending on whether the donors belong to *Bos taurus taurus* or *Bos taurus indicus* genetic groups [13,14]. Among representative *B. indicus* breeds, one finds Gir and Brahman livestock. The first is the main dairy cattle in tropical and subtropical regions [13], and the second is a long-life breed reared for meat production with adaptive traits in high ambient temperatures [15]. For *B. taurus* bovines, Holstein represents a widespread breed for dairy farming [16], while Romosinuano has been used in the meat industry and is the most important Creole Colombian breed, with the best adaptability, rusticity, fertility, meekness and hybrid vigor compared to other breeds [17].

Due to this, the objective of this study was to evaluate the presence of *AQP*, determine its mRNA levels in cumulus oocyte complexes (COC) of Brahman, Gir, Romosinuano, Holstein breeds and ♀Brahman × ♂Holstein, ♀Gir × ♂Holstein, ♀Holstein × ♂Gir, ♀Romosinuano × ♂Holstein and ♀Holstein × ♂Brahman embryos, and determine the genetic influence of breeds on expression patterns.

## 2. Materials and Methods

### 2.1. Follicular Aspiration of Donors

Bovine COC pool (*n* = 15 each) was taken by Ovum pick up (OPU) from nulliparous and non-lactating heifers aged between 48 to 96 months, belonging to the Brahman (*n* = 3), Gir (*n* = 3), Romosinuano (*n* = 3) and Holstein (*n* = 3) breeds, in Monteria–Córdoba, with an average temperature of 29 °C and relative humidity between 70% and 85%.

For the transvaginal follicular puncture, an ultrasound machine was used, equipped with a 5–7.5 MHz transvaginal probe and a 60 cm long OPU transvaginal device with a puncture guide and disposable puncture needle (20 G, 0.9 *70 mm) connected to a sterile 50 mL puncture tube through a Teflon hose. The OPU kit is completed with a vacuum pump calibrated at 50–53 mm Hg (20 mL/min). The follicular fluid obtained from the punctures was deposited in the puncture tube with the collection medium (lactated Ringer’s solution supplemented with fetal serum, 1% and sodium heparin, 5000 IU/L). The tubes were kept at 39 °C in a constant-temperature water bath. 

After aspiration, the COCs were classified according to Rodrigues et al. [18] in three grades (I, II, III), naked or cumulus-expanded depending on the homogeneity, cytoplasmic morphology, and cumulus cell compactness. For the purposes of this study, COCs of grade I, II, and III were used.

### 2.2. In Vitro Embryo Production

#### 2.2.1. In Vitro Maturation

COCs were washed four times in TALP-Hepes medium and once in in vitro maturation medium TCM199 supplemented with follicle-stimulating hormone and fetal bovine serum. About ten oocytes were then transferred in microdroplets of 50 µL of TCM199, for a period of 24 h at 38.5 °C, 5% CO_2_, and 95% humidity in the incubator.

#### 2.2.2. In Vitro Fertilization

Previously matured oocytes were washed four times in TALP-Hepes medium and transferred to TALPFert medium. Straws of 0.25 mL of semen were thawed in a water bath at 37 °C for 35 s, exposed to gradients of Percoll 45/90 in TALP-Sperm, then centrifuged at 500× *g* for 30 min, and the pellet was resuspended in TALPFert medium. This medium was incubated for 18 hours at 38.5 °C, 5% CO_2_, and 95% humidity.

#### 2.2.3. Embryo Culture

Zygotes were washed four times in Talp-Hepes medium, and then the embryos were transferred to 50 µL of culture medium SOF-BE1, where they remained in the blastocyst stage at 38.5 °C, 5% CO_2_, 5% O_2_, N_2_ balance and 95% humidity. Three embryo pools (*n*= 10 each one) were obtained for each ♀Gir × ♂Holstein, ♀Holstein × ♂Gir, ♀Brahman × ♂Holstein, ♀Holstein × ♂Brahman, and ♀Romosinuano × ♂Holstein cross.

### 2.3. RNA Extraction and cDNA Synthesis

Total RNA extraction was carried out from COC and embryo pools, using the RNA-solv reagent kit (OMEGA, Norcross, GA, USA), following the manufacturer’s instructions with modifications [13], and RNA quality was measured by spectrophotometry with the NanoDrop One (Thermo Scientific, Wilmington, DE, USA). cDNA synthesis was performed using the GoScript^TM^ Reverse Transcription System kit (Promega, Madison, WI, USA), and cDNA quality was determined through Endpoint PCR and agarose gel electrophoresis.

### 2.4. RT-PCR and Quantitative Polymerase Chain Reaction (qPCR)

RT-PCR amplification from cDNA was carried out using GoTaq® Flexi DNA polymerase (Promega, Madison, WI, USA) and *AQP* primer sets designed in Geneious Prime software (Table 1), following the manufacturer’s instructions for identifying the putative transcript expression of water channels in COC from four breeds and embryos from crosses. Amplification was realized in a ProFlex^TM^ PCR System (Applied Biosystems, Carlsbad, CA, USA) with modifications at annealing temperature for each set of primers (Table 1). Amplicons were revealed on 2% agarose gel electrophoresis (PowerPac™ HC, Bio-Rad, Hercules, CA, USA) stained with HydraGreen™ (ACTGene, Piscataway, NJ, USA) by using GeneRuler 100 bp DNA Ladder (Thermo Fisher Scientific, Waltham, MA, USA). Gel was visualized under UV light, in the ENDURO^TM^ GDS gel documentation system (Labnet International, Inc, Woodbridge, NJ, USA).

qPCR assays (*n* = 324) were run in duplicate with Luna® Universal qPCR Master Mix (New England BioLabs Inc.; Beverly, MA, USA) following the manufacturer’s instructions, in a QuantStudio 3 Real-Time PCR System (Thermo Fisher Scientific, Waltham, MA, USA) by Fast ramp program. Each primer product was validated by melt-curve analysis and gel electrophoresis imaging to ensure amplification from genomic DNA was not present. Relative gene expression was calculated using the 2^−∆∆Ct^ method [20], and *actb* was set as a normalization gene [19]. Data were expressed as fold change.

### 2.5. Statistical Analysis

Data were analyzed using descriptive statistics and the Shapiro–Wilk normality test on GraphPad Prism v9 (La Jolla, CA, USA). In the case of parametric data, an ANOVA test using Tukey’s test as *post hoc* was carried out. Otherwise, when the data were non-parametric, the Kruskal–Wallis test was performed using the Dunn *post hoc*. Differences were considered statistically significant with a *p*-value < 0.05.

## 3. Results

### 3.1. AQPs mRNA Level in COC 

**Figure 1:**
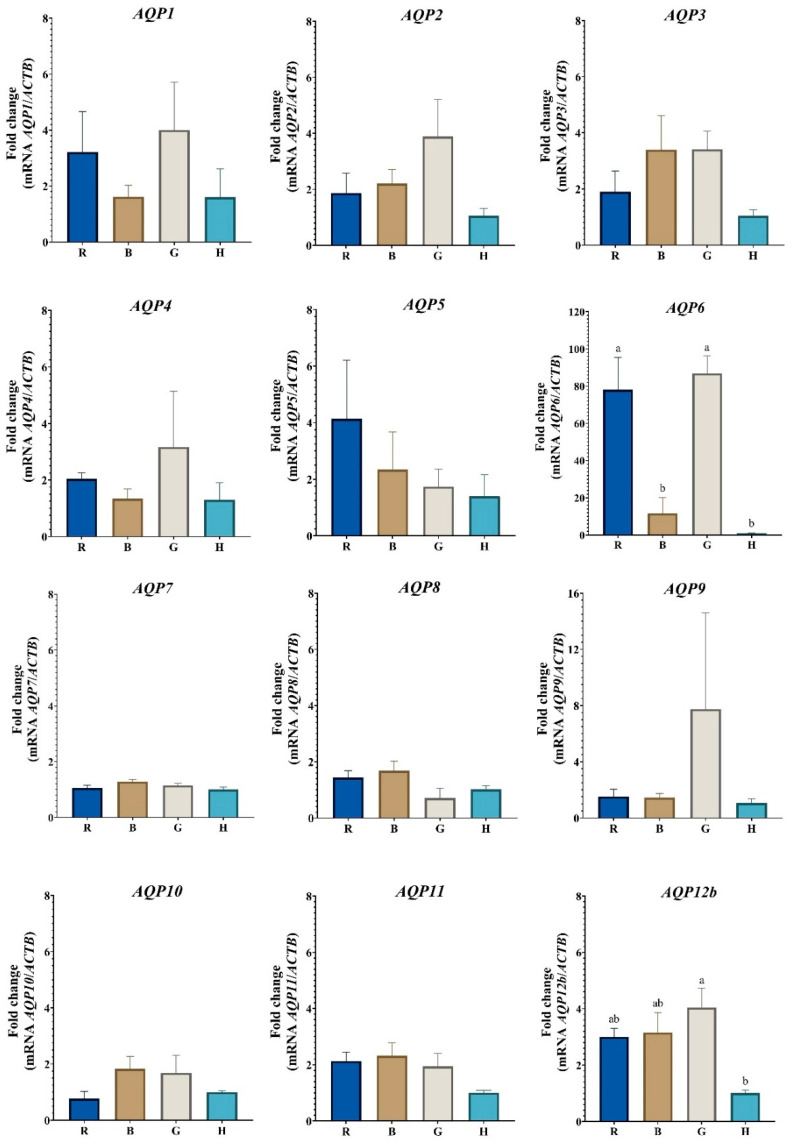
Relative expression of aquaporin gene transcripts in cattle COC from Romosinuano (R), Brahman (B), Gir (G), and Holstein (H) breeds. Different letters (a and b) denote statistical differences (*p* < 0.05). *AQPs* names in italics denote its mRNA expression levels.

In the case of COCs, statistical differences in the transcribed expression of AQP6 and AQP12b were found. Regarding AQP6, there are no differences in the level of transcripts between Romosinuano and Gir; however, *AQP6* mRNA expression was higher in Romosinuano compared to Brahman (*p* = 0.010) and Holstein (*p* = 0.004); likewise, Gir has a higher expression than Brahman (*p* = 0.004) and Holstein (*p* = 0.002) (Figure 1). In addition, the *AQP12b* mRNA level was higher in Gir compared to Holstein (*p* = 0.039), but this *AQP* was expressed similarly between Romosinuano, Brahman and Gir, and Romosinuano, Brahman and Holstein (Figure 1). Moreover, the expressions of *AQP1, AQP2, AQP3, AQP4, AQP5, AQP7, AQP8, AQP9, AQP10, AQP11* and *AQP12b* show no statistical differences in the COCs of the four breeds. 

On the other hand, despite the fact that no significant differences were determined in the expression of all the *AQPs* in the COCs, it is possible to see in Figure 1 that the Gir COCs show an upward numerical trend of the transcripts for all the *AQPs* (with the exception of *AQP5*) compared to the COCs of the other species, where the mRNA levels of *AQP2*, *AQP3*, *AQP7*, *AQP8*, *AQP10*, *AQP11* and *AQP12b* are higher in Gir and Brahman (*B. indicus* species), respectively, in contrast to those obtained by Romosinuano and Holstein, and the *AQP1, AQP4, AQP6* and *AQP9* transcripts have a higher expression in Gir and Romosinuano, respectively, unlike Brahman and Holstein.

### 3.2. AQPs mRNA Level in Cattle Embryos 

The expression of *AQP4* and *AQP9* in the blastocysts is influenced by the parental effect of Romosinuano as an oocyte donor breed, since the expression of these *AQPs* is similar in the crosses between ♀Gir × ♂Holstein and ♀Holstein × ♂Gir and between ♀Brahman × ♂Holstein and ♀Holstein × ♂Brahman, but differs between ♀Gir × ♂Holstein and ♀Romosinuano × ♂Holstein (*AQP4*, *p* = 0.04; *AQP9*, *p* = 0.01), with the mRNA level being lower in the latter. 

Moreover, AQP8 transcripts are related by the parental effect of Brahman as sire, since its expression differs in ♀Holstein × ♂Brahman compared to ♀Brahman × ♂Holstein (*p* = 0.0015), ♀Gir × ♂Holstein (*p* = 0.0016), ♀Holstein × ♂Gir (*p* = 0.0011) and ♀Romosinuano × ♂Brahman (*p* < 0.0001), but is similar to ♀Gir × ♂Holstein (*p >* 0.9999), ♀Holstein × ♂Gir (*p* = 0.9986) and ♀Romosinuano × ♂Brahman (*p* = 0.1818) when Holstein is the sire (♀Brahman × ♂Holstein).

**Figure 2:**
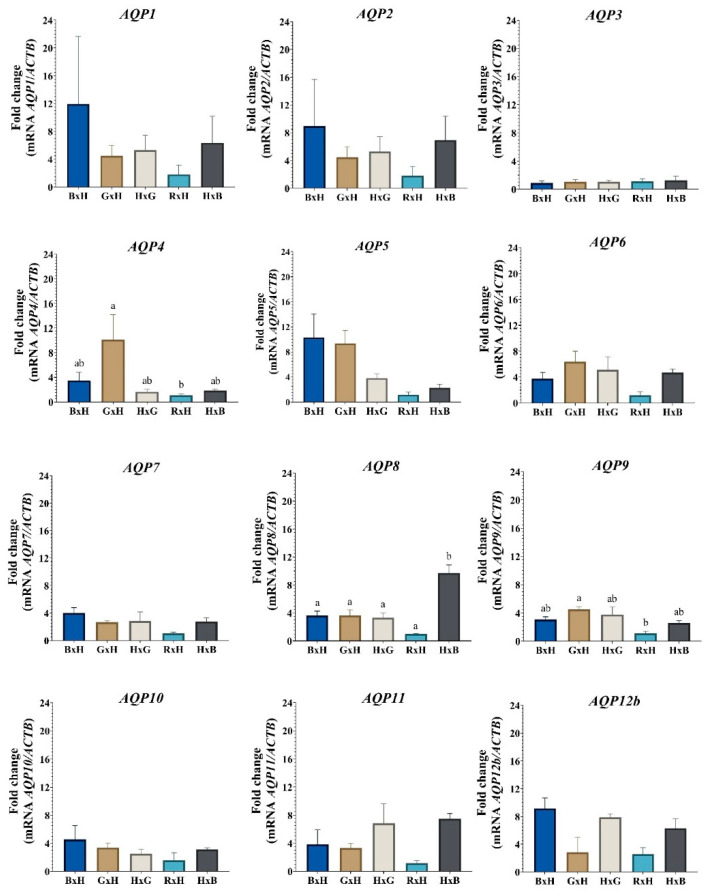
Relative expression of aquaporin gene transcripts in cattle embryos. **BxH**: ♀Brahman × ♂Holstein, **GxH**: ♀Gir × ♂Holstein, **HxG**: ♀Holstein × ♂Gir, **RxH**: ♀Romosinuano × ♂Holstein, and **HxB**: ♀Holstein × ♂Brahman. Different letters (a and b) denote statistical differences (*p* < 0.05). *AQPs* names in italics denote its mRNA expression levels.

On the other hand, in the expression of *AQPs* in the blastocysts of the five bovine crosses between the *B. indicus* and *B. taurus* breeds, it is possible to see, in Figure 2, the upward numerical trend in the *AQP1, AQP2, AQP5, AQP7, AQP10* and *AQP12b* transcripts when Brahman is the oocyte donor breed (♀Brahman × ♂Holstein), and a downward trend in the expression of all *AQPs* (except for *AQP3*) when Romosinuano is the oocyte donor breed (♀Romosinuano × ♂Holstein).

## 4. Discussion

### 4.1. AQPs mRNA Level in COC

In accordance with Jin et al. [12], bovine oocyte permeability occurs mainly by simple diffusion and, to a lesser extent, by *AQPs*. Therefore, we propose the importance of *AQP1*, *AQP10* and *AQP11* in the regulation of the flow of water, CO_2_, NH_3_ and glycerol through the cell membranes of the COCs of the Brahman, Holstein, Gir and Romosinuano breeds [21,22,23,24]. Since our study determined the expression of all the *AQPs* in the COCs of the Brahman, Holstein, Gir and Romosinuano breeds, and several studies have found the expression of *AQP1*, *AQP3*, *AQP4*, *AQP5*, *AQP7* and *AQP9* in bovine oocytes [12,25,26,27,28], their role in the development should be clarified.

Moreover, *AQP3* expression in the COC of *B. indicus* and *B. taurus* may play a role in water movement and cytoplasmic maturity in immature oocytes [12], influencing oocyte quality and its survival in cryopreservation [28,29]. On the other hand, the cryoprotectant ethylene glycol has been shown to stimulate the expression of *AQP7* in bovine oocytes [28,30]; thus, its presence in the COC of Gir, Romosinuano, Brahman and Holstein could be involved in tolerance to hyperosmotic stress during cryopreservation and a greater ease in the diffusion of water [28,30]. 

COCs are formed by undifferentiated granulosa cells (GC), and previous studies have characterized the presence of *AQP1, AQP3, AQP4, AQP5, AQP7* and *AQP9* in theca cells as GC of bovine ovarian follicles [25,31,32], this being consistent with *AQPs*’ expression in COCs of *B. indicus* and *B. taurus. AQP1, AQP5, AQP7* and *AQP9* may play a key role in the formation of the antrum in the Gir, Brahman, Holstein and Romosinuano breeds, since the transport of water and glycerol has been reported during the formation of the antrum mediated by *AQP1, AQP7* and *AQP9* [9,31], the involvement of *AQP3*, *AQP4,* and *AQP5* in antral follicular fluid flow [31,32], and the inhibition of GC apoptosis by all AQPs except *AQP4* and *AQP7* [9,33,34]. 

On the other hand, exposure to high temperatures affects folliculogenesis [35]. In this way, the adaptations to heat stress presented in *B. indicus* and *B. taurus* [36,37] may explain the absence of significant differences in the expression of the majority of the *AQPs*. The significant differences in the expression of *AQP6* and *AQP12b* may be related to the genetic diversity provided by migration, natural selection and geographical separation [38]. In addition, the higher expression levels of *AQP2*, *AQP3*, *AQP7*, *AQP10*, and *AQP12b* in Gir and Brahman may be due to their high adaptive capacity to harsh environments, given their tolerance to heat, and to both internal and external parasites, in comparison to Holstein and Romosinuano breeds [39,40].

To the authors’ knowledge, this study is the first report of *AQPs’* gene expression in the COC of Gir, Brahman, Romosinuano and Holstein, and of *AQP2*, *AQP6*, *AQP10* and *AQP12b* in bovine COC. 

### 4.2. AQPs’ mRNA Level in Embryos

In agreement with our study, the presence of *AQP3*, *AQP7*, *AQP9* and *AQP11* has been reported in bovine embryos by qPCR [11,13,41], and several bovine embryo transcriptome studies have found mRNA expression of all *AQPs*, except for *AQP10* [23,42,43,44,45,46]. Thus, *AQPs*’ expression in blastocysts of these breed crosses may be a heat-protection measure during embryonic development.

The function of *AQP1, AQP2, AQP8* and *AQP9* in the embryonic development of the five bovine crosses evaluated could be the homeostasis of the maternal-fetal fluid [47], since these *AQPs* have been reported in the fetal membrane, blastocysts and trophectoderm of different mammals, respectively [8,48,49,50,51]. Furthermore, it has been suggested that *AQP8* and *AQP9* could play a role in the preservation of cytoplasmic osmolarity during glycerol consumption in embryonic cells [52]. Moreover, *AQP9* and *AQP10* may be involved in the movement of other solutes, such as urea, purines and pyrimidines, during the embryo development of theses bovine crosses [3,8].

According to Wohlres–Viana et al. [13], no significant differences were found in the *AQP3* mRNA expression of the different embryo crosses between *B. indicus* and *B. taurus* breeds. Therefore, the presence of *AQP3* in these blastocysts could be related to fundamental developmental processes, such as the movement of water through the trophectoderm [8,53] and the maturation of the zygote to the morula stage by the facilitated diffusion of glycerol and ethylene glycol together with *AQP7* [49,54]. 

On the other hand, *AQP4* and *AQP5* have been found, as in this study, in *B. indicus* blastocysts and in 8-cell blastomeres and the blastocoel cavity of mice [50,55,56,57]. Therefore, their presence in the five bovine crosses between *B. indicus* and *B. taurus* could be related to water transport, given their high solute selectivity [55,58,59]. In addition, it has been found that *AQP5* is phosphorylated in the blastocyst stage, leading to its displacement to the cytoplasmic membrane in embryos in vivo [59], so it could play an important role in the general fluid homeostasis and blastocoel formation of the bovine crosses studied [57,58,59]. Likewise, *AQP6* may be involved in water transport and blastocoel formation of the bovine crosses given its high permeability to nitrate [57,60].

## 5. Conclusions

This is the first report on the twelve *AQPs* in the COCs of Gir, Brahman, Holstein and Romosinuano and in embryos of five crosses between *B. indicus* and *B*. *taurus*. Expression of the *AQP* mRNA level in COC and bovine embryos is consistent with previously described biological functions in other mammals. Our findings provide the basis for identifying key roles for *AQPs* in COCs and bovine embryonic development; however, further studies are required to characterize and elucidate their functions, as well as their possible use in cryopreservation.

## Figures and Tables

**Table 1:** Primer sequences for aquaporin (*AQP*) genes in cattle.

Gene	Primer sequence (5´-3´)		Primer Length (nt)	Tm (°C)	GC%	Amplicon Size (bp)	N° Accession/ Reference
*AQP1*	F	TCCTTCGGCTCCTCGGTGATTAC	23	66.4	57.0	174	NM_174702.3
	R	ATACTCCTCCACCTGACCGCTG	22	65.9	59.0		
*AQP2*	F	ACTCCGGTCCATAGCCTTCT	20	60.5	55.0	150	NM_001101199
	R	CCGATAGCCAGACCGAAG	18	58.4	61.0		
*AQP3*	F	CCTTATTGCTGGCCAGGTCTC	21	63.3	57.0	206	NM_001079794
	R	GGCCCGAAACAATAAGCTGGT	21	61.3	52.0		
*AQP4*	F	GGACTCAGCATCGCGACTATG	21	63.3	57.0	223	XM_027525836
	R	CGTGAACCGTGGTGACTCC	19	61.7	63.0		
*AQP5*	F	CATATGAACCCCGCCATCACG	21	63.3	57.0	165	XM_024992303
	R	CGCATTGACAGCCAGATTGC	20	60.5	55.0		
*AQP6*	F	GCTCTGTTTGCCGAGTTCCT	20	60.5	55.0	182	XM_002687279
	R	GTCACTGCAGGGTTGACGTG	20	62.5	60.0		
*AQP7*	F	GTCATATGGCAGAACGAGAA	20	56.4	45.0	151	NM_001076378
	R	CGAAGCCAAAACCCAAATTG	20	56.4	45.0		
*AQP8*	F	CAGAGGCAGCTGTATCCATG	20	60.5	55.0	157	NM_001206607
	R	CAGCCGATGAAGATGAACAG	20	58.4	50.0		
*AQP9*	F	GGACACTTTGGAGGAATCAT	20	56.4	45.0	143	NM_001205833
	R	CACTTCATCCGTCCAAAAAG	20	56.4	45.0		
*AQP10*	F	GGGCCAGGTTTCTCAGTTAC	20	60.5	55.0	162	XM_024989821
	R	CAGGGTAGGTGGCAAAGATG	20	60.5	55.0		
*AQP11*	F	AGACGGGTGCGATTAGACT	19	57.3	53.0	173	NM_001110069
	R	GACAGCCTCTATGATGACCG	20	60.5	55.0		
*AQP12b*	F	CTGACGTCTGCCTTCCTGAA	20	60.5	55.0	198	XM_005205063
	R	CCGGTACTTGCTCTTCTGAC	20	60.5	55.5		
*ACTB*	F	GGGATGAGGCTCAGAGCAAGAGA	23	63.6	56.5	118	[19]
	R	AGCTCGTTGTAGAAGGTGTGGTGCC	25	66.9	56.0		

Gene names in italics denote mRNA expression levels.

## Data Availability

The corresponding author will share the data provided in this study if requested.
